# Fiber ball white matter modeling in focal epilepsy

**DOI:** 10.1002/hbm.25382

**Published:** 2021-02-19

**Authors:** Lorna Bryant, Emilie T. McKinnon, James A. Taylor, Jens H. Jensen, Leonardo Bonilha, Christophe de Bezenac, Barbara A. K. Kreilkamp, Guleed Adan, Udo C. Wieshmann, Shubhabrata Biswas, Anthony G. Marson, Simon S. Keller

**Affiliations:** ^1^ Department of Pharmacology and Therapeutics Institute of Systems, Molecular and Integrative Biology, University of Liverpool UK; ^2^ Center for Biomedical Imaging Medical University of South Carolina Charleston South Carolina USA; ^3^ Department of Neuroscience Medical University of South Carolina Charleston South Carolina USA; ^4^ Department of Neurology Medical University of South Carolina Charleston South Carolina USA; ^5^ Department of Clinical Neurophysiology University Medicine Göttingen Göttingen Germany; ^6^ The Walton Centre NHS Foundation Trust Liverpool UK

**Keywords:** biomarkers, epilepsy, diffusion MRI

## Abstract

Multicompartment diffusion magnetic resonance imaging (MRI) approaches are increasingly being applied to estimate intra‐axonal and extra‐axonal diffusion characteristics in the human brain. Fiber ball imaging (FBI) and its extension fiber ball white matter modeling (FBWM) are such recently described multicompartment approaches. However, these particular approaches have yet to be applied in clinical cohorts. The modeling of several diffusion parameters with interpretable biological meaning may offer the development of new, noninvasive biomarkers of pharmacoresistance in epilepsy. In the present study, we used FBI and FBWM to evaluate intra‐axonal and extra‐axonal diffusion properties of white matter tracts in patients with longstanding focal epilepsy. FBI/FBWM diffusion parameters were calculated along the length of 50 white matter tract bundles and statistically compared between patients with refractory epilepsy, nonrefractory epilepsy and controls. We report that patients with chronic epilepsy had a widespread distribution of extra‐axonal diffusivity relative to controls, particularly in circumscribed regions along white matter tracts projecting to cerebral cortex from thalamic, striatal, brainstem, and peduncular regions. Patients with refractory epilepsy had significantly greater markers of extra‐axonal diffusivity compared to those with nonrefractory epilepsy. The extra‐axonal diffusivity alterations in patients with epilepsy observed in the present study could be markers of neuroinflammatory processes or a reflection of reduced axonal density, both of which have been histologically demonstrated in focal epilepsy. FBI is a clinically feasible MRI approach that provides the basis for more interpretive conclusions about the microstructural environment of the brain and may represent a unique biomarker of pharmacoresistance in epilepsy.

## Graphical abstract

Bryant et al. used fiber ball imaging to measure intra‐axonal and extra‐axonal white matter diffusion in patients with longstanding focal epilepsy. It is reported that extra‐axonal diffusion is different between patients with refractory and nonrefractory epilepsy and may represent a novel imaging biomarker of pharmacoresistance.

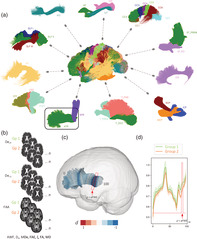

## 1 INTRODUCTION

Despite being classically considered to be a gray matter disorder, increasing evidence demonstrates cerebral white matter pathology in focal epilepsy. Epilepsy is a systems network brain disorder and is characterized by various neuropathological processes in the cerebral white matter, both in lesional and nonlesional cases (Concha, Livy, Beaulieu, Wheatley, & Gross, 2010; Deleo et al., 2018; Reeves et al., 2016). Identifying and characterizing these changes using noninvasive imaging techniques are important as this will provide deeper insights into the mechanisms of pathology in individual patients and potentially inform clinical and surgical decision‐making. For example, reconstruction of white matter structural networks and connectivity using diffusion MRI (dMRI) has been demonstrated to be an effective imaging biomarker of postoperative outcome in focal epilepsy (Bonilha et al., 2013; Bonilha, Jensen, et al., 2015; Keller et al., 2015; Keller et al., 2017). Diffusion tensor imaging (DTI) and extensions of which, such as diffusion kurtosis imaging (DKI), have been widely used to study white matter alterations in patients with focal epilepsy. Typical findings show a pattern of reduced fractional anisotropy (FA) and increased mean diffusivity (MD) in patients with focal epilepsy (Concha, Beaulieu, & Gross, 2005; Gross, Concha, & Beaulieu, 2006; McDonald et al., 2008), and a more widespread pattern of altered kurtosis metrics, suggesting an increased sensitivity of DKI in detecting WM abnormalities in epilepsy (Bonilha, Lee, et al., 2015; Glenn et al., 2016; Lee et al., 2014). However, while both DTI and DKI are reasonably sensitive to changes in white matter microstructure, these parameters only describe the tissue environment in terms of diffusion physics, rather than in biological parameters. They therefore lack microstructural specificity, making biological interpretation difficult (Fieremans, Jensen, & Helpern, 2011). To overcome this limitation, several diffusion methods have been proposed that more directly relate parameters with clear biological meanings to the diffusion MRI signal (Jelescu & Budde, 2017; Novikov, Kiselev, & Jespersen, 2018). These must necessarily introduce assumptions and approximations that explicitly connect water diffusion to microstructure. The simplest of such methods are based on the assumption that there are two nonexchanging water compartments within brain white matter: a fast (which models the less hindered extra‐axonal water) and a slow (which models the more restricted intra‐axonal water) diffusion compartment (Assaf, Freidlin, Rohde, & Basser, 2004; Fieremans et al., 2011; Kleban, Tax, Rudrapatna, Jones, & Bowtell, 2020).

The recently developed fiber ball imaging (FBI) is one such two‐compartment dMRI method (Jensen, Russell Glenn, & Helpern, 2016; Moss, McKinnon, Glenn, Helpern, & Jensen, 2019). FBI offers the ability to estimate the fiber orientation distribution function (fODF) of white matter fiber bundles along with related axon‐specific microstructural parameters. This is achieved by using strong diffusion weightings (b‐value > > 2000 s/mm^2^) to suppress the signal from the more mobile extra‐axonal water pool. Thus, by employing high b‐values FBI only needs to model the intra‐axonal compartment, a major advantage of FBI over other two‐compartment models. The main assumption of FBI is that water diffusion inside axons is accurately described by regarding axons as thin, straight, impermeable tubes. Compelling experimental evidence indicates that this is indeed a good approximation at least for myelinated axons (McKinnon, Jensen, Glenn, & Helpern, 2017; Moss et al., 2019; Veraart, Fieremans, & Novikov, 2019). Besides the fODF, FBI also allows estimation of the fractional anisotropy of the intra‐axonal space (FAA) and *ζ* (a property related to the axonal water fraction; AWF). Fiber ball white matter modeling (FBWM; McKinnon, Helpern, & Jensen, 2018) builds upon the principles of FBI but uses both high and low diffusion weightings to model the extra‐axonal space and the intra‐axonal space. FBWM therefore allows the estimation of additional microstructural parameters including the intrinsic intra‐axonal diffusivity (D_a_), the mean extra‐axonal diffusivity (MD_e_), the radial (D_e_,_⊥_) and axial (D_e,‖_) extra‐axonal diffusivity, the fractional anisotropy of the extra‐axonal space (FAE), and the AWF (Table 1). FBWM requires more data and more assumptions than FBI so that there is a natural division between the two. Specifically, FBWM is built by combining FBI and DKI data. Distinguishing FBI, DKI, and FBWM parameters emphasizes that they each have different biophysical underpinnings. FBI and FBWM have not previously been applied in patient cohorts.

**TABLE 1:** Diffusion parameters determined by FBI and FBWM, their biophysical meanings and potential biological interpretations

Parameters	Acronym	Meaning	Biological interpretation
Axonal water fraction (FBWM)	AWF	Amount of intra‐axonal water divided by the total amount of MRI‐visible water within a voxel Decreases if intra‐axonal water decreases, if extra‐axonal water increases (edema), or both	Potential loss of axons, edema
Intra‐axonal diffusivity (FBWM)	D_a_	Diffusivity of water molecules along the length of the axons	Changes in the intra‐axonal compartment (e.g., axonal beading)
Mean extra‐axonal diffusivity (FBWM)	MD_e_	Mean diffusivity of water within the extra‐axonal compartment. This compartment includes glial cells and extra‐cellular space	Analogous to MD but only for extra‐axonal water
Radial extra‐axonal diffusivity (FBWM)	D_e,⊥_	Radial diffusivity of the extra‐axonal compartment Perpendicular to the direction of highest extra‐axonal diffusivity	Analogous to RD but for extra‐axonal compartment
Axial extra‐axonal diffusivity (FBWM)	D_e,II_	Axial diffusivity of the extra‐axonal compartment in the direction of highest extra‐axonal diffusivity	Analogous to AD but for extra‐axonal compartment
Intra‐axonal fractional anisotropy (pure FBI)	FAA	The fractional anisotropy of just the water within of the intra‐axonal compartment Reflects the geometrical arrangement—if all axons in the same direction, this value is high	Analogous to regular FA, but typically has higher values as intra‐axonal water is more organized than extra‐axonal water
Extra‐axonal fractional anisotropy (FBWM)	FAE	Fractional anisotropy of just the water within extra‐axonal compartment	Analogous to regular FA but typically has lower values as extra‐axonal water is less organized than intra‐axonal water High values could represent tight packing of axons Parameter could be affected by myelination thickness
Zeta (pure FBI)	ζ	Equal to the AWF divided by the square root of the intra‐axonal diffusivity (D_a_) Scaled version of the AWF	Since D_a_ is often fairly constant across voxels, ζ is usually strongly correlated with AWF, but it is easier to measure

Abbreviations: AWF, axonal water fraction; FAA, fractional anisotropy of the intra‐axonal; FBWM, fiber ball white matter modeling; FAE, fractional anisotropy of the extra‐axonal.

**FIGURE 1:**
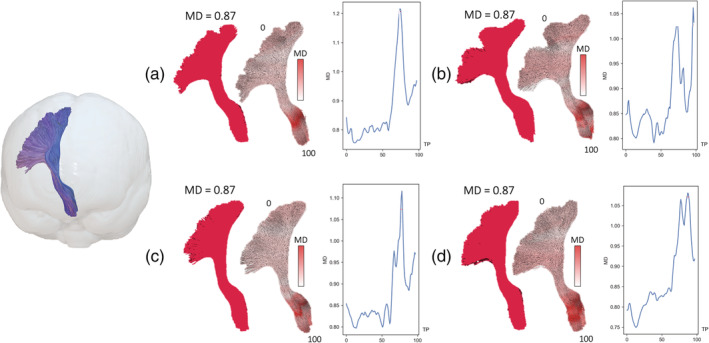
The advantage of assessing along‐tract diffusion metrics over whole tract averaging. (a–d) The left corticospinal tract (shown on the left in a glass‐brain projection for reference) of four subjects in the present study. Averaging a diffusion metric over the entire tract yields the same mean diffusivity (MD) value (0.87 μm^2^/ms) for each subject (full red tract). Analysis of MD at 100 points along the length of the corticospinal tract demonstrates considerable variability in regional MD within and between tracts. TP = tract points

Diffusion tractography provides the ability to reconstruct and assess diffusion characteristics averaged over whole white matter fiber bundles and along the longitudinal axis of tracts (along‐tract). Along‐tract analysis permits investigation of regionally specific alterations that may be more sensitive to regional pathology than averaging diffusion characteristics over entire tracts, particularly as tissue characteristics vary considerably along white matter tracts (Figure 1). This is particularly important for the in vivo assessment of neuropathology in neurological disorders as some pathological alterations occur in circumscribed regions of tracts, which may be overlooked when averaging diffusion metrics over entire tracts (Keller et al., 2017). Along‐tract approaches have demonstrated increased sensitivity for the identification of diffusion abnormalities in patients with temporal lobe epilepsy (Concha, Kim, Bernasconi, Bernhardt, & Bernasconi, 2012; Kreilkamp et al., 2019; Kreilkamp, Weber, Richardson, & Keller, 2017), high levels of sensitivity and specificity for the prediction of postoperative outcome (Keller et al., 2017), and greater yield of diffusion changes using DKI over conventional DTI (Glenn et al., 2016). The use of FBI and FBWM combined with along‐tract tractography offers the ability to investigate regional white matter tissue alterations with diffusion parameters that are biologically specific, which has not previously been possible using standard DTI approaches.

The modeling of several diffusion parameters with interpretable biological meaning may offer the development of new, noninvasive biomarkers of pharmacoresistance in epilepsy. It is unclear why 30% of patients with focal epilepsy do not attain seizure control despite anti‐epileptic drug (AED) treatment (Kwan & Brodie, 2000). The identification of a noninvasive imaging biomarker of treatment outcome would allow earlier stratification to more personalized treatment methods. However, there have been limited insights into the mechanisms and markers underlying pharmacoresistance using advanced MRI methods. Markers of hippocampal neuronal‐glial changes have been associated with pharmacoresistance in patients with temporal lobe epilepsy using MR spectroscopy (Campos et al., 2010; Pimentel‐Silva et al., 2020). Other studies have reported increased white matter and gray matter volumetric changes in patients with refractory relative to nonrefractory focal epilepsy using morphometric techniques applied to T1‐weighted images (Bilevicius et al., 2010; Kim, Kim, Lee, & Park, 2017), which may relate to differences in functional connectivity between refractory and nonrefractory patients (Pressl et al., 2019). However, characterization of diffusion properties between patient groups using advanced methods that offer biological interpretation has not, to our knowledge, been performed. DTI and other traditional diffusion imaging methods provide indirect information about the underlying cellular microstructure. Diffusion metrics such as FA and MD represent the diffusion dynamics of all underlying compartments mixed together. Biophysical models, such as FBWM, attempt to separate the diffusion dynamics into different compartments (i.e., intra‐axonal and extra‐axonal) to get a more specific understanding of changes happening in the underlying microstructure. This strategy is particularly useful when studying disease processes that affect microstructural compartments differently and could provide novel insights into pharmacoresistance in epilepsy.

In the present study, we have investigated intra‐axonal and extra‐axonal white matter diffusion alterations in patients with focal epilepsy by combining FBI and FBWM with an along‐tract tractography approach. By specifically recruiting patients with either refractory or nonrefractory focal epilepsy, we sought to determine whether changes in the microscopic white matter environment may be a marker of pharmacoresistance. We expected to find differences between patient groups in FBI and FBWM metrics, which have a more easily understandable biological basis compared to standard DTI.

## 2 METHODS

### 2.1 Participants

Fifteen adults with focal epilepsy who had responded well to AED treatment (mean age = 41 years, *SD* = 12, eight female; hereon referred to as nonrefractory epilepsy), 15 adults with focal epilepsy who had refractory epilepsy (mean age = 38 years, *SD* = 12, eight female) and 15 healthy controls (mean age = 38 years, *SD* = 13, seven female) were recruited into this study. Patients with epilepsy were recruited from outpatient clinics and clinical databases from the Walton Centre NHS Foundation Trust in Liverpool, UK. All patients and controls gave informed consent and ethical approval was given by the Health Research Authority (UK Research Ethics Committee [REC] ID = 17/NW/0342; IRAS project ID = 220138). Focal epilepsy was diagnosed by expert epileptologists based on seizure semiology and inter‐ictal EEG recordings acquired in context of standard clinical care. Nonrefractory patients had experienced no seizures for at least 6 months prior to recruitment into the study. Refractory patients were experiencing persistent seizures prior to recruitment. Patients with primary generalized seizures, nonepileptic seizures, previous neurosurgery or known progressive neurological disease were excluded from this study. A breakdown of all demographic and clinical information for patients is provided in Table 2.

**TABLE 2:** Patient characteristics

Study ID	Group	Age	Sex	Sfree	Type	Lat	Loc	Onset	Dur	Familial history	Birth complications	FC	Known neurological issues	Radiological MRI findings
FBIP02	Re	32	M		CFTB	R	T	0	32	N	N	Y	Meningitis when baby	None
FBIP03	Re	33	M		SFTB	L	F	16	17	N	N	N		None
FBIP04	Re	21	F		F	R	F	1.5	19.5	N	Emergency c‐section	Y		None
FBIP05	Re	34	M		F	U	F	13	21	N	N	N		Fluid effusion seen in the mastoid air cells, more on the right side
FBIP06	Re	18	M		CF	L	F	10	8	N	N	N		Scattered T2 white matter focal hyperintensities, seen mainly in the subcortical white matter of the frontal lobes bilaterally
FBIP07	Re	51	M		F	U	T	14	37	N	N	Y	Encephalitis when infant	Increased T2 signal with loss of volume of the body and tail of the hippocampi bilaterally. Likely bilateral mesial temporal sclerosis
FBIP08	nRe	26	M	2	SFTB	U	U	24	2	N	N	N		None
FBIP09	nRe	48	F	3	SFTB	R	T	12	36	N	N	N		Pineal cyst measuring about 1.2 cm in maximum dimension, a few scattered tiny foci of T2 hyperintensity seen in the subcortical and deep white matter of the frontal lobes bilaterally. Mega cisterna magna (incidental)
FBIP10	Re	50	F		CFTB	U	U	18	32	N	N	N		Slight upward convexity of the upper margin of the pituitary (incidental)
FBIP11	Re	32	M		CFTB	R	T	23	9	N	N	N		Quadrigeminal plate lipoma, mega cisterna magna (incidental)
FBIP12	Re	51	F		CF	R	T	11	40	Y	N	N		Slightly increased T2 hyperintensity in right hippocampus, subtle hippocampal volume asymmetry. Suspicion of right mesial temporal sclerosis
FBIP13	nRe	24	F	3		R	T	21	3	N	N	N		Temporal horn of the right lateral ventricle slightly more prominent compared to the left; no evidence of mesial temporal sclerosis
FBIP14	Re	36	F		CFTB	U	U	20	16	N	N	N		Borderline loss of cerebellar volume
FBIP15	Re	38	F		CFTB	L	F	17	21	Y	N	N		Lesion seen at the posterior aspect of the left frontal lobe; mild generalized loss of cerebellar volume
FBIP16	Re	48	F		CFTB	U	T	10	38	N	N	N		None
FBIP17	Re	41	F		CFTB	U	U	27	14	N	Premature	N		Focal encephalomalacia/gliosis seen in the left lateral orbital gyrus, anterior aspect of the left temporal lobe; borderline loss of cerebellar volume
FBIP18	nRe	33	M	1	CFTB	U	U	29	4	N	N	N		10 mm × 6 mm dural‐based structure seen at the left posterior temporal/occipitotemporal region; appearances compatible with meningioma
FBIP19	Re	28	M			R	T	13	15	N	N	N		Right hippocampus sclerosis
FBIP20	nRe	46	F	1.5	CFTB	U	U	22	24	N	N	N		Upward convexity of the upper margin of the pituitary (incidental)
FBIP21	nRe	60	M	1	SFTB	U	R	56	4	Y	N	N		None
FBIP22	nRe	38	F	5	SFTB	R	T	30	8	U	U	N/K	Poss encephalitis—not confirmed	None
FBIP24	Re	58	F		SFTB	U	U	11	47	N	U	N		Right‐sided subependymal gray matter heterotopia along ventricular trigone and temporal and occipital horns of lateral ventricle Polymicrogyria extending from the periventricular heterotopic gray matter Additional closed lip schizencephaly and dysplasia Right‐sided cerebellar volume loss. Cerebellar atrophy
FBIP25	nRe	44	M	5	SFTB	R	T	39	5	N	N	N	Car accident when 15, brain injury	Encephalomalacia/gliosis in the left gyrus rectus and medial orbital gyrus. Focal damage in right temporal pole. Small focus of gliosis/encephalomalacia is close to left temporal bone. Mild periventricular T2 hyperintensity adjacent to the left ventricular trigone/supratrigonal white matter
FBIP26	nRe	26	M	2	CFTB	U	T	21	5	Y	N	N		Right hippocampus marginally smaller than the left
FBIP27	nRe	29	F	3	CF	U	U	22	7	N	N	N		None
FBIP28	nRe	35	M	2	CFTB	R	F	31	4	N	N	N	Accident when 11, brain injury	Encephalomalacia/gliotic changes in right frontal lobe extending to left frontal lobe. Some anterior corpus callosum volume loss. Gliotic/encephalomalacia changes in the left parietal lobe. Subtle volume loss of right temporal pole
FBIP29	nRe	59	F	4.5	SFTB	U	U	54	5	N	3 months premature	Y		Mild‐to‐moderate small vessel ischaemia
FBIP30	nRe	54	M	11	SFTB	L	T	43	11	N	Spina bifida, hydocephalus	Y	Hydrocephalus when born	Right‐sided frontal porencephalic cyst with surrounding gliotic changes Smaller porencephalic cyst in the left frontal lobe. Atrophy of the anterior aspect of the body of the corpus callosum Mild enlargement of the lateral and third ventricles secondary white matter volume loss.
FBIP31	nRe	51	F	3	SFTB	L	F	48	3	U	U	N		Gliosis of anterior left superior frontal gyrus Mild generalized brain volume loss. Mild small vessel type ischaemic changes in white matter adjacent to anterior aspect of right lateral ventricle
FBIP32	nRe	38	F	0.75	CFTB	U	U	36	2.5	Y	N	N	Enlarged ventricles	Heterotopic subependymal gray matter nodules seen along the body of the right lateral ventricle

Abbreviations: CF, complex focal seizures; CFTB, complex focal to bilateral seizures; Dur, duration of epilepsy; F, focal seizures (unknown whether simple or complex); FC, febrile seizures; FH, family history of epilepsy; Fr, frontal; L, left; Lat, lateralization (left / right); Loc, localization (lobar); N, no; nRe, nonrefractory; Re, refractory; R, right; SF, simple focal seizures; SFree, number of years seizure free; SFTB, simple focal to bilateral seizures; T, temporal; U, unresolved; Y, yes.

### 2.2 MR acquisition

All patients and controls were scanned at the Liverpool Magnetic Resonance Imaging Centre (LiMRIC) using a 3 T Siemens Prisma MR scanner. Scanning included a T1‐weighted magnetization‐prepared rapid acquisition gradient echo sequence (MPRAGE; 192 slices, repetition time [TR] = 2,000 ms, inversion time [TI] = 912 ms, echo time [TE] = 2.25 ms, resolution = 1.0 × 1.0 × 1.0 mm^3^, flip angle = 8°, acquisition time = 7:30 min). In order to calculate FBI and FBWM parameters, two diffusion‐weighted scans were performed: (a) an FBI sequence (TR = 4,400 ms, TE = 100 ms, 46 axial slices, resolution = 2.7 × 2.7 × 2.7 mm^3^, b‐values = 0, 5,000 s/mm^2^, 128 directions) to calculate ζ and FAA; (b) a DKI sequence that was parameter‐matched to the FBI scan (b‐values = 0, 1,000, 2000 s/mm^2^, 30 directions) which, along with the FBI scan, allows for the calculation of D_a_, MD_e_, D_e,⊥_, D_e,II_, FAE, and AWF. A standard DKI scan was also performed in order to calculate standard DTI metrics of FA and MD (TR = 3,200 ms, TE = 90 ms, 50 axial slices, resolution = 2.5 × 2.5 × 2.5 mm^3^, b‐values = 0, 1,000, 2,000 s/mm^3^, 64 directions).

### 2.3 MR preprocessing

dMRI data were preprocessed for scalar map generation (FA, MD, AWF, D_a_, FAE, MD_e_, D_e,II_, D_e,⊥_, FAA, ζ; Table 1) using Matlab Version R2016b. Preprocessing included MRtrix3 denoizing and Gibbs ringing removal (Tournier et al., 2019) and correction for Rician noise biases. FMRIB Software Library (FSL; http://fsl.fmrib.ox.ac.uk/fsl/fslwiki/FSL;Smith et al., 2004) was used to extract and skull‐strip a nondiffusion weighted (i.e., b = 0) image to generate a “b0” mask. The b0 mask was binarized and used in FSL eddy current correction to mitigate effects of image artifacts (Andersson & Sotiropoulos, 2016) and motion correction, with the b‐vectors rotated based in the rigid‐body alignment parameters (Leemans & Jones, 2009). DTI (FA and MD) parameter maps were generated using diffusional kurtosis estimator (DKE; https://www.nitric.org/projects.dke/; Tabesh, Jensen, Ardekani, & Helpern, 2011) from the standard DKI scan. FBI (FAA and ζ) and FBWM (AWF, D_a_, MD_e_, D_e,⊥_, D_e,II_, and FAE) maps were generated using in‐house Matlab scripts (https://github.com/m-ama/FBWM/) using the FBI and FBI‐matched DKI scans, as previously described (McKinnon et al., 2018). To prepare data for white matter fiber tract segmentation, eddy‐corrected diffusion images, b0 masks, and b‐value and b‐vector files were all normalized to standard Montreal Neurological Institute (MNI) space using FSL FLIRT (Greve & Fischl, 2009; Jenkinson, Bannister, Brady, & Smith, 2002; Jenkinson & Smith, 2001) and the MNI FA template available in FSL.

### 2.4 White matter fiber tract segmentation

**FIGURE 2:**
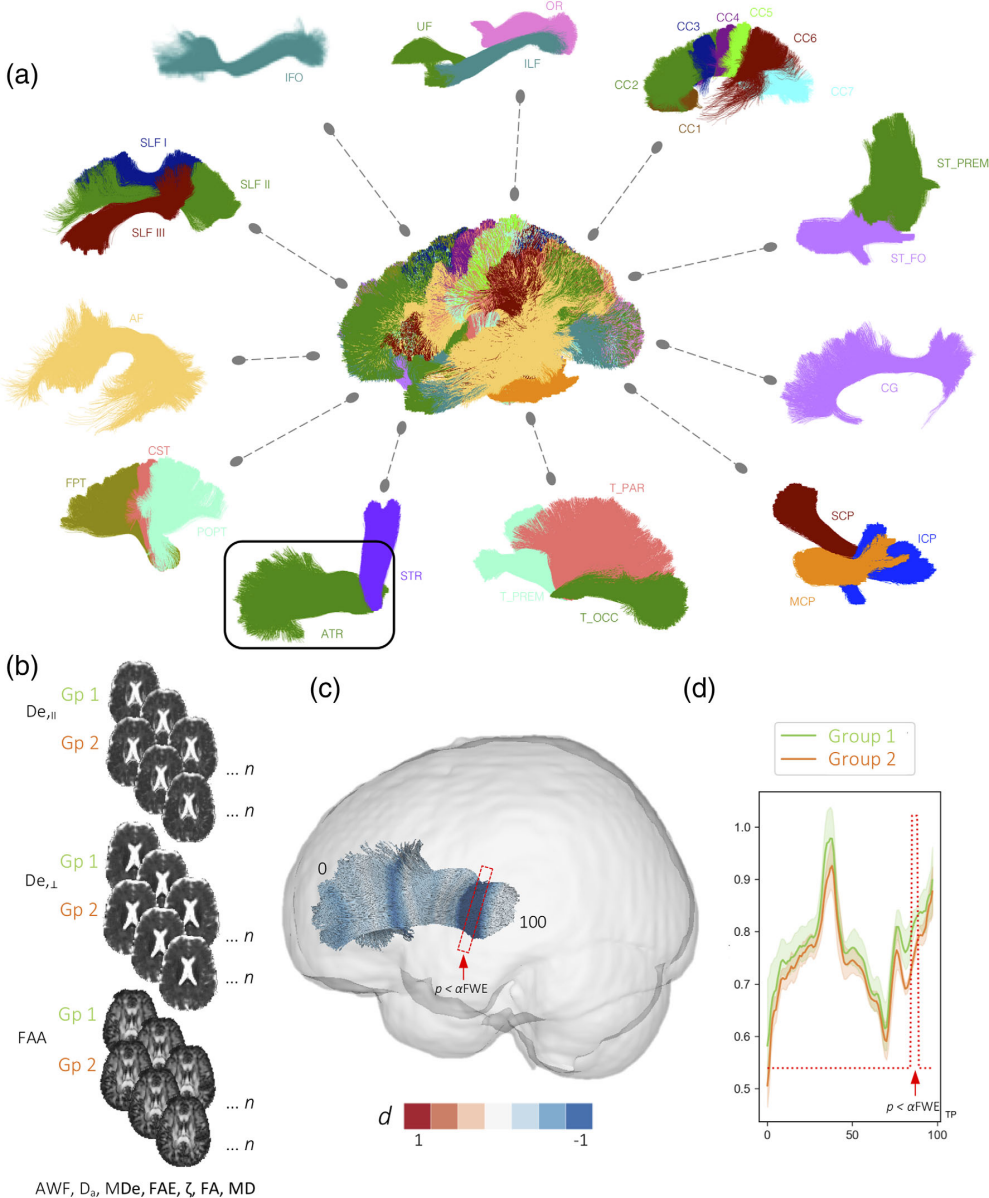
(a) White matter tracts automatically segmented using TractSeg included in the present study. See Table 3 for definition of abbreviations. (b) Illustration of group comparisons of FBWM maps for along‐tract analyses. (c) Visualization of Cohen's d effect sizes computed along the length of an exemplar tract (ATR; see a) that is significantly different between two groups. (d) Mean (±SEM) diffusion values are plotted for each point along the tract. *P* < *α*FWE: region along the tract where the *p* value is smaller than the *α*FWE and considered statistically significant. TP = tract points

Fifty white matter tracts were generated using TractSeg (https://github.com/MIC-DKFZ/TractSeg/) (Wasserthal, Neher, Hirjak, & Maier‐Hein, 2019; Wasserthal, Neher, & Maier‐Hein, 2018) (Figure 2a and Table 3). TractSeg is a recently developed method for WM tract segmentation, which shortens typical tractography pipelines by using a fully convolutional neural network. This method directly segments white matter tracts in fields of fODF peaks via a fully convolutional neural network and has been demonstrated to yield more accurate tract segmentation compared to six other reference methods of tractography and tract segmentation (Wasserthal et al., 2018). Tractometry, the final part of the TractSeg pipeline, outputs diffusion parameter values for 100 points along each segmented white matter fiber tract which we used for along‐tract analysis. The 100 points for each tract were standardized across participants so that direct comparisons for each FBI and FBWM map could be made between groups (Figure 2b–d).

**TABLE 3:** White matter tracts analyzed in the present study; see Figure 2

AF	Arcuate fascicle
ATR	Anterior thalamic radiation
CC1	Corpus callosum: Rostrum
CC2	Corpus callosum: Genu
CC3	Corpus callosum: Rostral body, premotor
CC4	Corpus callosum: Anterior midbody, primary motor
CC5	Corpus callosum: Posterior midbody, primary somatosensory
CC6	Corpus callosum: Isthmus
CC7	Corpus callosum: Splenium
CG	Cingulum
CST	Corticospinal tract
FPT	Frontopontine tract
ICP	Inferior cerebellar peduncle
IFO	Inferior occipitofrontal fascicle
ILF	Inferior longitudinal fascicle
MCP	Middle cerebellar peduncle
OR	Optic radiation
POPT	Parieto‐occipital pontine
SCP	Superior cerebellar peduncle
SLFI	Superior longitudinal fascicle segment I
SFLII	Superior longitudinal fascicle segment II
SLFIII	Superior longitudinal fascicle segment III
STR	Superior thalamic radiations
ST_FO	Striatofrontal orbital
ST_PREM	Striatoprefrontal
T_PAR	Thalamoparietal
T_PREM	Thalamopremotor
T_OCC	Thalamo‐occipital
UF	Uncinate fascicle

### 2.5 Statistical analysis

TractSeg permitted statistical analysis of FBI and FBWM parameter values along 100 points of each white matter tract and compared across three groups: controls, patients with refractory focal epilepsy and patients with nonrefractory focal epilepsy. Statistical analysis along‐tracts was performed as per the tractometry documentation available online,^1^ which is based on the statistical analysis approach described by Yeatman, Dougherty, Myall, Wandell, and Feldman (2012). This method uses a permutation based multiple comparison correction to appropriately adjust *p*‐values given the correlation structure of the data (Nichols & Holmes, 2002). We adopted this approach and corrected for age, sex, and multiple comparisons using family wise error correction. Results were considered significant if the minimal *p*‐value for each along‐tract point was lower than the alpha value corrected for multiple comparison (Yeatman et al., 2012). Cohen's d effect sizes were calculated for diffusion parameters and corrected for small sample size. Effect sizes were plotted along the length of tracts showing statistically significant differences between groups (Figure 2c).

## 3 RESULTS

There was no significant difference between refractory and nonrefractory groups in the number of patients with (refractory = 9, 60%; nonrefractory = 10, 67%) and without (refractory = 6, 40%; nonrefractory = 5, 33%) radiologically diagnosed lesions that were considered to be not incidental (Table 2; *χ* ^2^ = 0.14, *p* = .70). There was no significant difference in age (*t* = 0.62, *p* = .54), age of onset of epilepsy (*t* = 0.02, *p* = .99) or duration of epilepsy (*t* = 0.71, *p* = .54) between refractory and nonrefractory patient groups.

**FIGURE 3:**
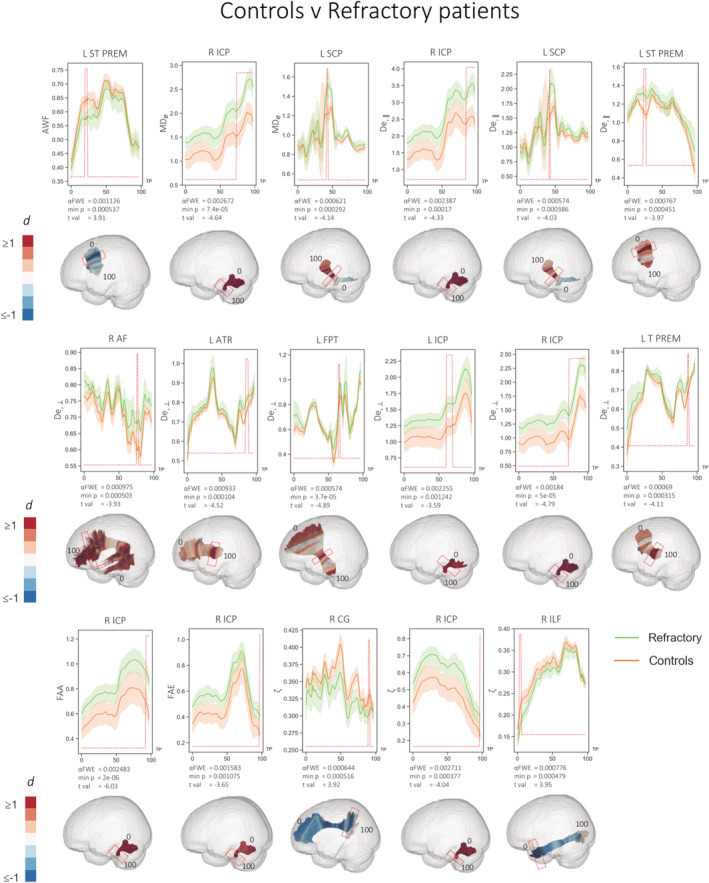
Statistically significant (red peaks) along‐tract differences in FBWM diffusion properties in refractory patients compared to controls. 3D plots show Cohen's d values projected onto the relevant tracts with significant region highlighted by red boxes. Effect sizes greater than and equal to 1 are shown in dark red, with those less than and equal to −1 shown in dark blue. Significantly different ζ in regions of the right SLF III and right UF not illustrated (see Table 4). Diffusivities are expressed in units of μm^2^/ms, while ζ values are in units of ms^1/2^/μm; all other quantities are dimensionless. TP = tract points

All significant differences in FBI and FBWM parameters between patients with refractory epilepsy, nonrefractory epilepsy and controls are tabulated in Table 4. Significant differences between patients with refractory epilepsy and controls are illustrated in Figure 3. Significant differences were observed for seven diffusion measures over 12 tracts and effect sizes for significantly different regions were large (d = ±1 or greater). Inspection of the along‐tract values for both groups demonstrate that refractory patients showed consistent diffusion alterations over the entire length of tracts and statistical analysis revealed only the peak difference between groups. In putative measures of intra‐axonal diffusivity, patients with refractory epilepsy had significantly reduced AWF in a region of the left striatoprefrontal tract (ST PREM) and reduced ζ in regions of the right cingulum (CG), right inferior longitudinal fascicule (ILF), right superior longitudinal fascicle segment III (SLF III), and right uncinate fascicle (UF) relative to controls. Increased ζ was seen in a region of the right inferior cerebellar peduncle (ICP). There were more widespread changes in putative measures of extra‐axonal diffusivity, including increases in MD_e_ and D_e,II_ in regions of the right ICP, left superior cerebellar peduncle (SCP), and in the left ST PREM in D_e,II_ only. Additional D_e,⊥_ increases were observed in the right arcuate fascicle (AF), left anterior thalamic radiation (ATR), left frontopontine tract (FPT), and left and right ICP. Significantly increased FAA and FAE was also observed within the right ICP in refractory patients compared to controls.

**TABLE 4:** Fiber ball white matter modeling (FBWM) significant differences between patients with refractory epilepsy, nonrefractory epilepsy and controls

Parameters	Tract	Controls	Refractory	αFWE	Min *p*‐value	t‐value	Cohen's d
AWF	L ST PREM	0.56 (0.04)	0.50 (0.07)	0.001125	.000537	−3.91	1.39
FAE	R ICP	0.18 (0.10)	0.26 (0.10)	0.002152	.001075	3.65	−1.30
MD_e_	R ICP	0.97 (0.20)	1.39 (0.45)	0.002295	7.40E‐05	4.64	−1.65
	L SCP	1.17 (0.13)	1.45 (0.23)	0.000558	.000292	4.14	−1.47
D_e,II_	R ICP	1.30 (0.26)	1.82 (0.61)	0.002143	.00017	4.34	−1.54
	L SCP	1.76 (0.26)	2.22 (0.39)	0.000665	.000386	4.03	−1.43
	L ST PREM	1.36 (0.10)	1.56 (0.20)	0.001006	.000451	3.97	−1.41
D_e,⊥_	R AF	0.74 (0.05)	0.83 (0.08)	0.000969	.000503	3.93	−1.40
	L ATR	0.77 (0.03)	0.82 (0.05)	0.001134	.000104	4.52	−1.61
	L FPT	0.71 (0.05)	0.79 (0.07)	0.000745	3.70E‐05	4.89	−1.74
	L ICP	0.71 (0.08)	0.87 (0.19)	0.002178	.001242	3.59	−1.28
	R ICP	0.81 (0.18)	1.17 (0.39)	0.00254	5.00E‐05	4.79	−1.70
	L T PREM	0.74 (0.02)	0.79 (0.05)	0.000724	.000315	4.11	−1.46
FAA	R ICP	0.28 (0.14)	0.48 (0.07)	0.002887	2.00E‐06	6.03	−2.14
ζ	R CG	0.32 (0.02)	0.29 (0.02)	0.000618	.000516	−3.92	1.39
	R ICP	0.15 (0.08)	0.21 (0.05)	0.002866	.000377	4.04	−1.44
	R ILF	0.24 (0.03)	0.19 (0.03)	0.000944	.000479	−3.95	1.40
	R SLF III	0.39 (0.02)	0.36 (0.02)	0.000781	.000219	−4.24	1.51
	R UF	0.23 (0.03)	0.18 (0.03)	0.001022	.000909	−3.71	1.32
		Controls	Nonrefractory				
D_a_	L STR	2.10 (0.27)	2.50 (0.31)	6.00E‐04	.000565	−3.89	1.38
D_e,II_	R T PAR	1.18 (0.05)	1.30 (0.10)	0.000451	.000163	−4.35	1.55
	R T OCC	1.21 (0.06)	1.37 (0.13)	0.000484	.000171	−4.33	1.54
D_e,⊥_	L ATR	0.76 (0.03)	0.85 (0.06)	0.000758	3.00E‐06	−5.81	2.06
	L T PREM	0.69 (0.03)	0.75 (0.04)	0.000754	.000199	−4.28	1.52
FA	CC1	0.37 (0.04)	0.31 (0.05)	0.002028	.000699	3.81	−1.35
FAA	R STR	0.61 (0.02)	0.65 (0.03)	0.000539	.000229	−4.23	1.50
MD	L ATR	0.91 (0.05)	0.98 (0.04)	0.000683	.000113	−4.48	1.59
		Refractory	Nonrefractory				
MD_e_	R CST	1.11 (0.07)	1.02 (0.05)	0.001903	.001867	3.44	−1.22
	R ICP	1.36 (0.13)	1.17 (0.21)	0.001822	.000732	3.79	−1.35
D_e,II_	R ICP	1.86 (0.20)	1.63 (0.25)	0.003316	.001363	3.56	−1.26
D_e,⊥_	L FPT	0.79 (0.05)	0.71 (0.03)	0.000809	5.60E‐05	4.74	−1.69
	R ICP	1.11 (0.17)	0.94 (0.23)	0.002599	.000789	3.76	−1.34
FA	R SCP	0.44 (0.05)	0.37 (0.05)	0.003208	.001382	3.55	−1.26
FAA	L OR	0.63 (0.03)	0.67 (0.03)	0.000264	.000237	−4.21	1.50
	L T OCC	0.62 (0.02)	0.67 (0.03)	0.000253	.00011	−4.50	1.60
MD	R CST	1.14 (0.08)	1.01 (0.08)	0.00058	1.10E‐05	5.33	−1.89
	L FPT	1.04 (0.05)	0.93 (0.05)	0.000512	1.90E‐05	5.15	−1.83
	R ICP	1.13 (0.09)	1.04 (0.12)	0.003937	.002445	3.33	−1.18
	L POPT	1.08 (0.08)	0.96 (0.08)	0.000644	.00013	4.43	−1.58
	R POPT	1.22 (0.11)	1.05 (0.10)	0.000544	8.40E‐05	4.59	−1.63
ζ	MCP	0.44 (0.05)	0.36 (0.07)	0.005229	.001876	3.43	−1.22
	R SCP	0.32 (0.05)	0.26 (0.06)	0.003404	.001244	3.59	−1.28

*Note*: The mean (*SD*) of the individual diffusion parameter, minimum *p*‐value and its corresponding *α* value, *t* statistic, and Cohen's *d* for the peak of each significant tract are provided. Diffusivities are expressed in units of μm^2^/ms, while ζ values are in units of ms^1/2^/ μm; all other quantities are dimensionless. Abbreviations for parameters are provided in Table 1 and for tracts in Table 3.

**FIGURE 4:**
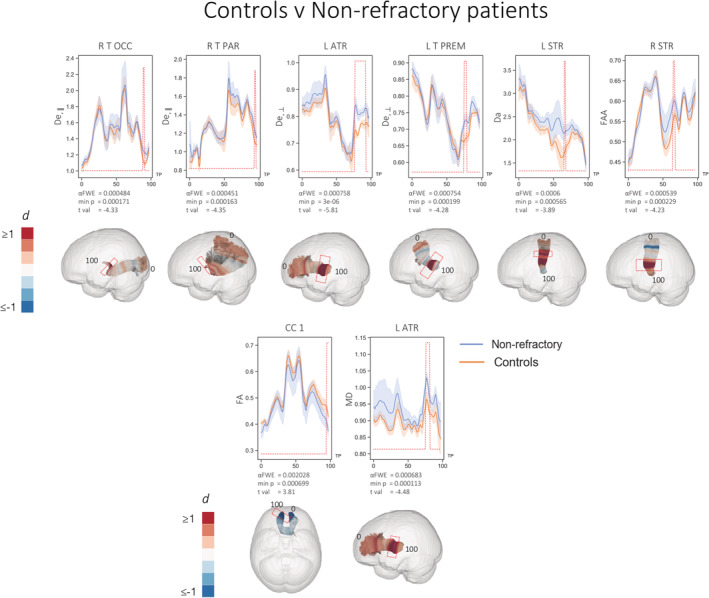
Statistically significant (red peaks) along‐tract differences in FBWM diffusion properties in nonrefractory patients relative to controls. 3D plots show Cohen's d values projected onto the relevant tracts with significant region highlighted by red boxes. Effect sizes greater than and equal to 1 are shown in dark red, with those less than and equal to −1 shown in dark blue. Diffusivities are expressed in units of μm^2^/ms, while ζ values are in units of ms^1/2^/μm; all other quantities are dimensionless. TP = tract points

Significant differences in FBI and FBWM parameters between patients with nonrefractory epilepsy and controls are illustrated in Figure 4. Patients with nonrefractory epilepsy showed substantially fewer diffusion alterations and smaller effect sizes. Significant differences were observed for six diffusion measures over six tracts. Patients had significantly increased values of the following parameters compared to controls: D_a_ in a region of the left superior thalamic radiations (STR), D_e,II_ of the right thalamoparietal (T PAR) and right thalamo‐occipital (T OCC) tracts, increased D_e,⊥_ of the left ATR and left thalamopremotor (T PREM) tracts, and FAA of the right STR.

**FIGURE 5:**
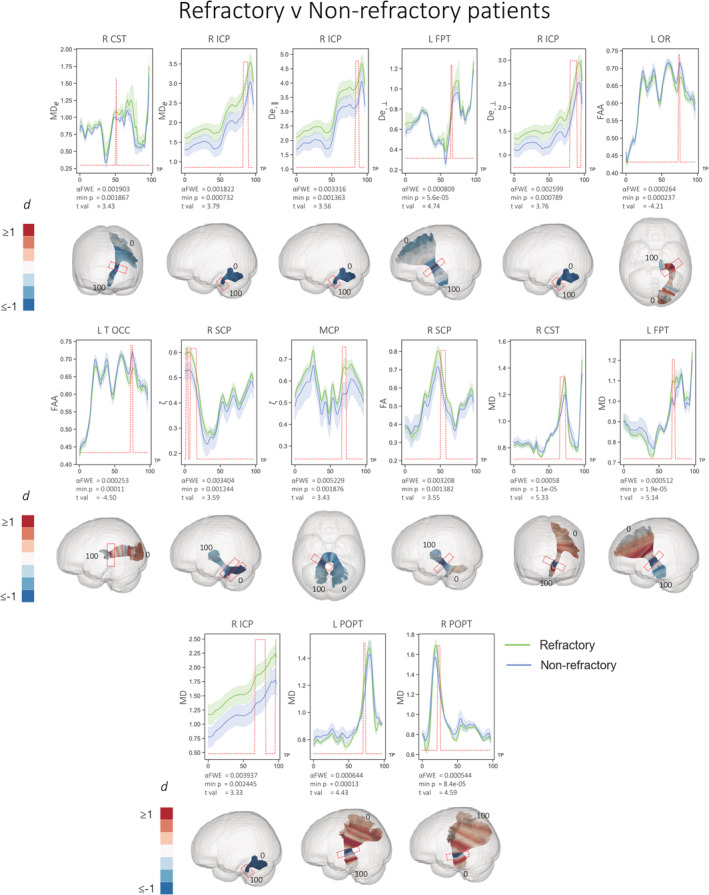
Statistically significant (red peaks) along‐tract differences in FBWM diffusion properties in refractory patients relative to nonrefractory patients. 3D plots show Cohen's d values projected onto the relevant tracts with significant region highlighted by red boxes. Effect sizes greater than and equal to 1 are shown in dark red, with those less than and equal to −1 shown in dark blue. Diffusivities are expressed in units of μm^2^/ms, while ζ values are in units of ms^1/2^/μm; all other quantities are dimensionless. TP = tract points

In direct comparisons between patient groups, patients with refractory epilepsy showed evidence of significantly increased putative measures of extra‐axonal diffusivity compared to patients with nonrefractory epilepsy (Figure 5). These were manifest as significantly increased MD_e_ in regions within the right corticospinal tract (CST) and right ICP, increased D_e,II_ in right ICP, and increased D_e,⊥_ in regions of the left FPT, right and right ICP. Patients with refractory epilepsy also had significantly increased ζ in regions of the middle cerebellar peduncle (MCP) and right SCP. Significantly reduced FAA was observed in regions of the left optic radiation (OR) and left T OCC in refractory patients.

Analysis of conventional DTI metrics showed limited and more spatially restricted diffusion changes. There were no significant differences in FA or MD between refractory patients and controls. Nonrefractory patients had significantly decreased FA in the rostrum of the corpus callosum (CC1) and increased MD in a region of the left ATR compared to controls (Figure 4). Direct comparisons between patient groups revealed significantly reduced FA in nonrefractory patients in the right SCP and increased MD in refractory patients in regions of the right CST, left FPT, right ICP, left and right parieto‐occipital pontine tracts (POPTs) (Figure 5).

## 4 DISCUSSION

This study is the first to apply FBI and FBWM to a clinical population. We have used this advanced dMRI method together with along‐tract tractography to investigate white matter microstructural changes in patients with longstanding focal epilepsy. By using FBI and FBWM, we are able make inferences about the biological processes underlying tissue diffusion changes, which has previously been difficult to do using conventional DTI. As expected, we found differences between patient groups and controls, and in accordance with previous literature we found increases in diffusivity parameters in patient groups. Relative to controls, refractory patients had significant increases in the putatative extra‐axonal measures of MD_e_, D_e,II_, and D_e,⊥_ across several WM tracts. Conversely, patients with nonrefractory epilepsy had limited statistically significant increases in extra‐axonal diffusion measures compared to controls; this contrast in extra‐axonal diffusion between patient groups was exemplified in direct comparisons, which demonstrated significantly greater measures of extra‐axonal diffusion in refractory patients. Analysis of FBI and FBWM diffusion measures was substantially more revealing than conventional DTI metrics. We discuss the biological and clinical significance of these findings before highlighting pertinent methodological issues.

### 4.1 Biological and clinical implications

The FBI/FBWM two compartment model permits separation of intra‐axonal and extra‐axonal water diffusion. MD_e_ is a measure of extra‐axonal diffusivity, which is similar to MD but only for the extra‐axonal diffusion compartment. Similarly, D_e,⊥_ is analogous to RD using conventional DTI, but for the extra‐axonal compartment. An increase in extra‐axonal space has been argued to reflect an increase in neuroinflammation via microglial activation (Pasternak et al., 2012; Schwartz, Butovsky, Bruck, & Hanisch, 2006). Neuroinflammation is known to have an important role in epilepsy (Amhaoul, Staelens, & Dedeurwaerdere, 2014). Neuroinflammation has been studied extensively using positron emission tomography (PET), specifically using the radioligands (R)‐[11C]PK11195 and 18F‐PBR111 as measures of translocator protein (TSPO), which is upregulated in the context of activated glial cells (Scott, Mahmud, Owen, & Johnson, 2017). PET‐derived measures of neuroinflammation have been validated in rat models of TLE—kainic acid‐induced status epilepticus—where increased TSPO expression has been associated with increased microglial activation determined using immunohistochemistry (Amhaoul et al., 2015). In people with well circumscribed and phenotyped focal epilepsy, neuroinflammatory changes have been demonstrated in regions beyond the presumed seizure focus. For example, increased TSPO uptake has been demonstrated in temporal and extra‐temporal lobe regions, ipsilateral and contralateral to the presumed seizure focus, in patients with TLE (Gershen et al., 2015; Hirvonen et al., 2012). Increased neuroinflammatory changes have been reported in a case study of post‐ictal human focal epilepsy using PET (Butler et al., 2016). Interestingly, previous work has demonstrated increased uptake of (R)‐[11C]PK11195 in rats with persistent seizures despite phenobarbital treatment compared to rats that responded well to treatment (Bogdanovic et al., 2014). Taken together, these findings suggest that neuroinflammatory changes in local and distal brain regions are associated with refractory seizures, which may be considered consistent with the widespread extra‐axonal water diffusion changes observed in the present study. Given that we preferentially observed increased extra‐axonal diffusion in refractory patients, we suggest that imaging measures sensitive to the extra‐axonal space may be a biomarker of pharmacoresistance. However, in human epilepsy studies, it is difficult to discern whether increased extra‐axonal space and/or neuroinflammatory processes are a cause or consequence of active seizures. Nevertheless, it is interesting that patients with nonrefractory epilepsy who had not experienced seizures for an average of over 3 years (Table 2) also showed evidence of more restricted pattern of extra‐axonal diffusion compared to controls (Figure 4).

An alternative explanation is that increased extra‐axonal space is directly due to axonal degeneration (Rodriguez‐Cruces & Concha, 2015). Reduced myelin content and axonal density of myelinated and nonmyelinated axons has been reported in refractory focal epilepsy (Garbelli et al., 2012), and reduced cumulative axonal membrane circumference is associated with reduced FA in WM tracts in close proximity to the epileptogenic zone (Concha et al., 2010). Reduced axonal density inherently generates increased extra‐axonal space (Rodriguez‐Cruces & Concha, 2015), and this process could be driving increased extra‐axonal diffusion in patients with epilepsy. Most studies examining axonal density in refractory focal epilepsy naturally restrict analysis to the presumed epileptogenic zone (given the opportunity to perform histological studies on resected specimens). Whether the same neuropathology extends to axons extending distally across the brain as we have shown here is a more contentious issue. We report extra‐axonal diffusivity alterations across many WM tracts that are presumably not part of the epileptogenic zone. The underlying histological cause of these changes remain unknown.

We additionally report increases in regional white matter MD in patients with refractory epilepsy compared to those with nonrefractory epilepsy. MD is a measure of all diffusion which is directionally averaged and may in itself be a marker of cellularity, edema and necrosis (Alexander et al., 2011). Increased MD is often reported in many dMRI studies of epilepsy (Concha, Beaulieu, Collins, & Gross, 2009; Jiang et al., 2017; McDonald et al., 2008). Moreover, MD was found to be superior to other conventional diffusion metrics in predicting postoperative seizure outcome from preoperative DTI in patients with TLE (Keller et al., 2017). The results presented here suggest that the previously widespread observed changes in WM diffusivity using conventional DTI in patients with refractory focal epilepsy are largely driven by the extra‐axonal diffusivity component.

### 4.2 Methodological considerations

While FBI and FBWM are recently developed techniques that had not been previously applied to clinical populations, other dMRI two−/multicompartment models have been employed in clinical contexts to further understand the cerebral microstructural environment. Free‐water imaging approaches have been proposed for detecting neuroinflammatory markers in disorders known to have neuroinflammatory components including schizophrenia (Pasternak et al., 2012), Parkinson's disease (Planetta et al., 2016), and concussion (Pasternak et al., 2014). Neurite orientation dispersion and density imaging (NODDI) (Zhang, Schneider, Wheeler‐Kingshott, & Alexander, 2012) has been used to demonstrate alterations in intracellular volume fraction, a marker of neurite density, in malformed brain regions causing epilepsy (Winston et al., 2014) and in widespread temporal and extratemporal brain regions in TLE (Winston et al., 2020). Other work has reported that NODDI‐derived orientation dispersion index maps reveal cortical and subcortical abnormalities in patients with nonlesional focal epilepsy (Rostampour, Hashemi, Najibi, & Oghabian, 2018). Multicompartment models are better able to understand the biological processes driving changes in diffusion parameters (Winston et al., 2020). By modeling diffusion with two−/multicompartment models, evidence suggests that improved detection of focal abnormalities and further insights into the underlying microstructure of the brain is possible. While these models have provided important insights into microstructural brain changes associated with neuropathology, there are notable issues with many dMRI tissue models. These include problems with nonlinear fitting algorithms, on which many of these models are based, which can lead to increased computational times, sensitivity to noise and imaging artifacts, and issues related to the accuracy of the underlying idealizations (Novikov et al., 2018). FBWM mitigates these difficulties with a two‐compartment model based on well‐supported assumptions and a simple computational scheme involving a straightforward linear transformation of the dMRI signal together with a one‐dimensional numerical optimization. Further, FBWM improves substantially upon the white matter tract integrity (WMTI) method (Fieremans et al., 2011). The WMTI method (Fieremans et al., 2011) assumes that all axons in a given voxel are approximately aligned in the same direction, which creates issues in regions of crossing fibers. FBWM overcomes this by using the fODF from FBI rather than assuming a particular geometric arrangement of axons and, as such, should more accurately model regions with crossing fibers.

Rather than averaging diffusion metrics over each reconstructed white matter tract we chose to utilize an along‐tract approach. Given that white matter tissue diffusional characteristics vary long the length of tracts, it is advantageous to capture this variation in clinical studies as averaging across whole tracts may obscure regionally specific and pathologically relevant diffusion alterations (Figure 1). This is especially important for the development of imaging markers of treatment outcome in epilepsy; only by quantifying diffusion changes along tracts did a previous study identify preoperative markers of excellent and suboptimal postoperative seizure outcomes in patients with refractory TLE (Keller et al., 2017). In the present study, we were able to identify the circumscribed region of principle difference in extra‐axonal diffusivity within tracts between patient groups and controls. Moreover, given the inconsistent regional differences in diffusion metrics within tracts between refractory and nonrefractory patients (Figure 5), we would be unable to identify significant differences in tract characteristics if diffusion measures were averaged over the entire tracts. In order to further improve upon this method, future work could make use of the fODFs generated directly from FBI. These are expected to yield improved tractography results over fODFs from standard DTI (Jensen et al., 2016; Moss et al., 2019). Our data suggest that dMRI two‐compartment models analyzed over the length of tracts may be a noninvasive marker of pharmacoresistance in patients with epilepsy. It is possible to use alternative image analysis techniques to examine microstructural properties of white matter pathways, such as tract‐based spatial statistics (TBSS). TBSS offers the opportunity to perform voxel‐based analyses of representative regions while minimizing multiple comparisons by evaluating voxels constrained to a core white matter skeleton in standard space. However, we chose to apply a tractography approach that offers increased patient‐constrained neuroanatomical precision and permits comprehensive profiling of microstructural properties along full white matter tracts, which we suggest may provide the best opportunity to identify individualistic diffusion‐based biomarkers of treatment outcome (Keller et al., 2017). Finally, despite that this application of FBI and FBWM modeling is the first in a clinical cohort and has demonstrated robust extra‐axonal alterations in patients recruited according to treatment response, it has done so in a modest sized cohort. Further insights into treatment response and patient cognitive profiles may be achieved in larger prospective studies.

## 5 CONCLUSIONS

This is the first clinical application of FBI and FBWM. The two‐compartment diffusion model has provided the means to separate intra‐axonal and extra‐axonal diffusion alterations in patients with longstanding epilepsy. The strongest findings were with parameters sensitive to extra‐axonal diffusion, which may suggest significant neuroinflammatory processes and/or axonal degeneration. This work demonstrates that these changes are more widespread in patients with refractory epilepsy compared to those with well‐controlled seizures located along eloquent white matter tracts not considered to be part of the epileptogenic zone in patients with focal seizures.

## CONFLICT OF INTERESTS

The authors declare no potential conflict of interest.

## ETHICS STATEMENT

All patients and controls gave informed consent and ethical approval was given by the Health Research Authority (UK Research Ethics Committee [REC] ID = 17/NW/0342; IRAS project ID = 220138).

## Data Availability

The data that support the findings of this study are available on request from the corresponding author. The original data are not publicly available due to ethical restrictions.

## ENDNOTE

^1^
https://github.com/MIC-DKFZ/TractSeg/blob/master/resources/Tractometry_documentation.md
